# Feline breeding and pregnancy management: What is normal and when to intervene

**DOI:** 10.1177/1098612X221079708

**Published:** 2022-02-25

**Authors:** Bodil Ström Holst

**Affiliations:** Department of Clinical Sciences, Faculty of Veterinary Medicine and Animal Sciences, Swedish University of Agricultural Sciences, Uppsala, Sweden

**Keywords:** Pregnancy, parturition, mastitis, pyometra

## Abstract

**Practical relevance:**

Cats are common pets worldwide. Successful breeding of cats starts with the selection of suitable breeding animals, and care should be taken to avoid inbreeding. Keeping cats in smaller groups reduces stress and facilitates management.

**Clinical challenges:**

Breeding cats is challenging in many ways. Group housing is a common scenario, and care should be taken not to have groups that are too large, because of the risk of stress and infectious diseases. Feline pregnancy and parturition both vary in length, which is one reason why it may be challenging to diagnose dystocia. In queens with pyometra, a vaginal discharge may not be evident due to their meticulous cleaning habits.

**Audience:**

This review is aimed at clinicians in small animal practice, especially those in contact with cat breeders.

**Patient group:**

Reproductive emergencies occur in both intentionally and unintentionally bred cats, and more often in young or middle-aged queens. Pyometra tends to be a disease of older queens.

**Evidence base:**

Evidence is poor for many conditions in the breeding queen, and information is extrapolated from the dog or based on case reports and case series.

**Figure:**
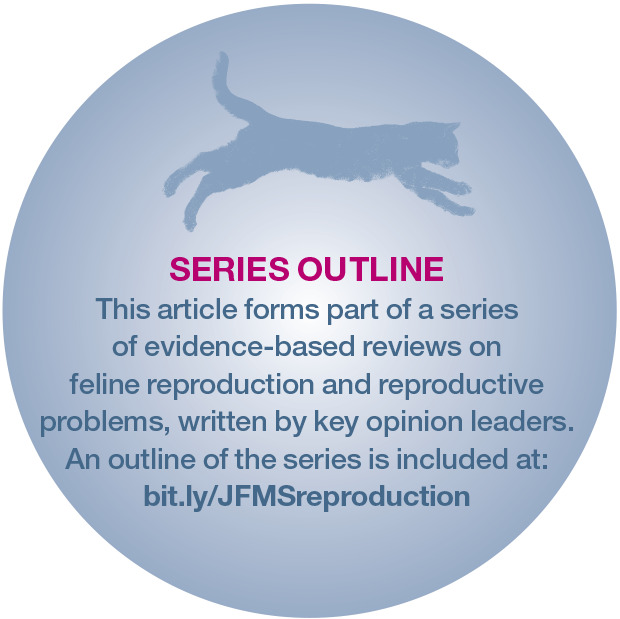


## Introduction

In contrast to unplanned reproduction, which leads to the global problem of millions of unwanted cats, planned breeding generally gives rise to well cared for, and much appreciated, pets. A cornerstone in ethical and sustainable breeding is not to cause suffering to the dam or offspring. To ensure this, knowledge of traits carried by the dam and possibly transmitted to the offspring is needed. In reality, such knowledge is often difficult to acquire; hence, judging suitability for breeding is challenging. Several diseases cannot be tested for in advance, and the decision as to whether the origin of a condition in an individual case is genetic or not is often not clear-cut.

Most modern cat breeds have been developed during the past 150 years, with emphasis on appearance, such as hair length, coat colour and coat pattern. Breeding for special traits has led to selective inbreeding, and extensive use of certain popular males, with the result that several cat breeds show a high degree of inbreeding.^1^ If too few individuals meet the requirements for breeding, negative effects related to inbreeding may be a result. Inbreeding reduces genetic variability and increases homozygosity, leading to genetic diseases, malformations and a negative effect on reproductive performance. Teratospermia in cats has been associated with reduced genetic diversity.^2^ in dogs, inbreeding has been linked to decreased litter size and an increased proportion of stillborn pups.^3^ Inbreeding has also been associated with a small litter size in cats.^4^

When selecting breeding animals, traits such as health, temperament and reproductive performance are important. Breeding must involve not only the ‘best’ individuals, but also individuals that are ‘good enough’, to ensure that there is a sufficient number of breeding animals to maintain the genetic diversity within the breed. Advice from the practising veterinarian should inform the selection of animals suitable for breeding.

## Infectious disease and housing of breeding animals

Infectious diseases are a constant threat to breeders. Many feline infectious agents may give rise to subclinical infections and, unless identified, subclinical carriers may constitute a persistent source of infectious agents in the cattery. Upper respiratory tract disease, including conjunctivitis, is a common problem in catteries.^5^ Agents causing upper respiratory tract disease are transmitted via aerosol and direct contact, and transmission is thus favoured by group housing.

The number of cats that are kept in a cattery may vary with region and breed, but a mean of three to five intact females per cattery has been described in Italy and Sweden.^5,6^ Many breeders keep at least one breeding male, and it is not uncommon to also keep older, castrated cats.^5^ With larger groups the risk of stress among cats increases, and it is also difficult to manage any infections that occur. Ideally, a cattery with many cats should maintain smaller groups. With a group size of three or four cats, the number of animals that need to be tested and possibly treated in the case of an infectious disease outbreak is manageable, and stress levels for this size of group have been described as being similar to those of single-living cats.^7^

With breeding comes the introduction of kittens to the group, and they are more prone than adult cats to developing clinical disease. Typically clinical disease is associated with excretion of large numbers of infectious agents (in secretions, during sneezing, etc). Infected kittens are thus a problem in themselves, and they also contribute to increased transmission rates. Stress may lead to activation of subclinical infections, although this is not always associated with clinical disease. Feline herpesvirus-1 (FHV-1) isa typical example; excretion of FHV-1 occurs within weeks of administration of corticosteroids.^8^ Reproduction can likewise be stressful. Increased blood cortisol concentrations have been described during lactation,^9^ and FHV-1-infected queens have been demonstrated to shed virus 2–10 weeks after parturition.^8^

**Figure 1:**
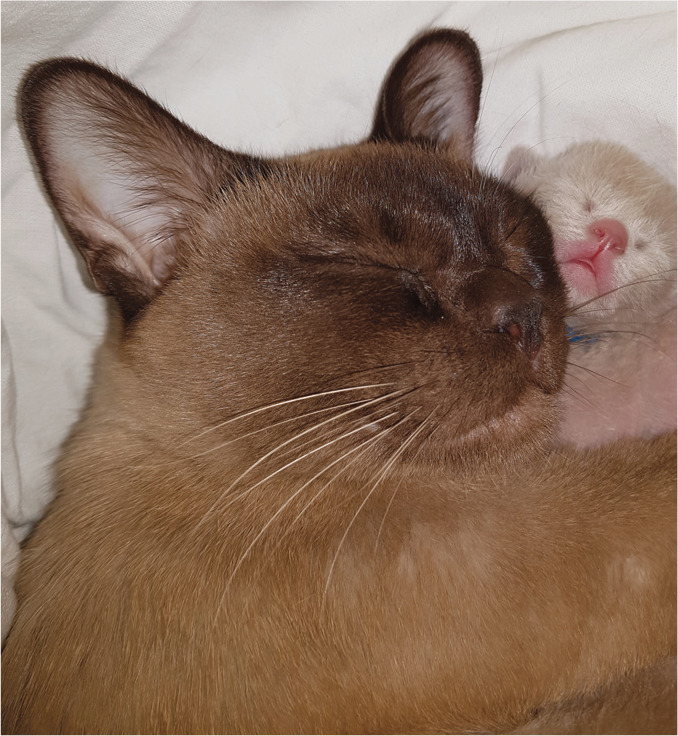
The queen is a major source of infections for the kittens. *Courtesy of Ulrika Hermansson*

Keeping the pregnant queen separated from the other cats during pregnancy, at least for the last 2–3 weeks (covering the incubation period of most infections), reduces the risk of the female getting infected by them and developing clinical disease, thus reducing the risk of infecting the offspring (Figure 1). After parturition, keeping the queen with kittens separate from other cats in the cattery or household further protects the kittens from disease. Although other cats in the group may not show clinical signs of disease, they may carry and excrete infectious agents such as FHV-1, *Chlamydia* species, *Mycoplasma* species and feline calicivirus (FCV), and thus pose a risk to unvaccinated kittens.

Infectious agents may be introduced with new cats or with resident cats that have been away - for example, for breeding or to cat shows. To reduce the risk of introducing infections into the cattery, cats should be kept in quarantine, in a separate room or separate building, for 2–3 weeks. During quarantine clinical signs may appear - either because of stress leading to reactivation of an existing infection or because of a newly acquired infection – and the infected cat can be properly taken care of before joining the group. In addition, cats can be tested during quarantine to avoid the introduction of infectious agents, including intestinal protozoa. However, it should be noted that certain agents, for example *Tritrichomonas foetus*, may be difficult to detect in infected cats that do not show any clinical signs.

Most infections are managed with husbandry practices that aim to reduce stress and minimise the risk of transmission. Some agents, such as feline leukaemia virus and feline immunodeficiency virus, can be specifically tested for before mating.^10^ For feline infectious peritonitis (FIP), a genetic susceptibility has been shown,^11,12^ and a difference in disease prevalence between breeds has been described.^13,14^ However, when positive genetic selection was attempted among laboratory cats, resistance to the disease decreased rather than increased, associated with increased homozygosity.^15^ This illustrates the complexity of FIP. Even though, as a rule, close relatives of a cat that has developed FIP should be avoided for breeding, they may be used where necessary to maintain heterozygosity and, in turn, the health of the population.

## Blood typing

In cats, the AB blood group system consists of three types: A, B and AB. in an individual cat, the blood type can be checked with immuno-haematological (serological) methods or by genotyping.^16^ Blood group incompatibility may cause neonatal isoerythrolysis (Ni) in kittens, a potentially fatal condition that may be seen when a type B queen is bred with a type A tom. As discussed in the accompanying review on fading kitten syndrome in this series, Ni arises because of the presence of naturally occurring alloantibodies: cats with type A or B blood possess alloantibodies against the blood type antigen they lack. in particular, type B cats will have high titres of alloantibodies against type A (anti-A isoagglutinins). When the kittens of a type B queen and type A tom ingest colostrum during the first hours after birth, ingested antibodies, including alloantibodies, will be transferred to the circulation, and may, depending on the amount and their affinity, cause clinical disease. Clinical signs include icterus and pigmenturia, anaemia, failure to thrive and sudden death.

The prevalence of different blood types varies between regions and breeds.^17^ Blood typing of the male and female may thus be relevant before mating, especially in breeds with a high proportion of blood group B (eg, British Shorthair, Devon Rex and Birman), and hence an increased risk of Ni. if a type B queen has been mated with a type A male, Ni in kittens can be avoided if they are prevented from suckling, using a stocking or similar, during the period of colostral uptake of immunoglobulins from the gut (ie, the first 16 h^18^). After this time, there is no uptake of colostrum (and thus anti-A isoagglutinins) through the gut. Depriving kittens of colostrum leads to lack of transfer of passive immunity, but does not necessarily lead to increased kitten mortality,^19^ especially if the environment is free of pathogens and not stressful. Administration (subcutaneous or intraperitoneal) of serum from an adult cat can correct igG deficiency in colostrum-deprived kittens, but to avoid Ni, type B serum should not be given to type A kittens.^20^

## Pregnancy and pregnancy diagnosis

Cats should be in good condition when mated. The energy requirement of the dam increases continuously during pregnancy, by approximately 10% per week; by the end of pregnancy the queen’s energy intake should be 25–50% above maintenance levels.^21^ However, care should be taken to avoid the cat being overweight, as an association between obesity and both dystocia and the number of stillbirths has been described.^22^ Routine deworming practices in a cattery will depend upon relevant national legislation and individual risk assessments. A single treatment of pregnant queens with emodepside spot-on approximately 7 days before expected parturition is recommended to prevent lactogenic transmission of *Toxocara cati* larvae to the kittens.^23^ An alternative approach to deworming the queen is to treat the young kittens (eg, with benzimi-dazoles or pyrantel pamoate).

Endocrine changes during pregnancy include elevated concentrations of progesterone, which is not only produced by the corpora lutea but also by the placenta.^24^ Progesterone concentrations increase during the first 3 weeks, reach a plateau and start decreasing after approximately 5 weeks.^25^ Although concentrations decrease towards parturition, basal concentrations may not be reached until after parturition,^26^ and progesterone concentrations cannot be used to predict parturition in cats. After an ovulation not resulting in pregnancy (pseudopregnancy), concentrations increase to a lesser degree, and decrease to reach low concentrations after 40–45 days, although mildly elevated concentrations may persist until after day 62.^26^

Oestradiol concentrations are low during the majority of the gestation period, but increase in the last week before parturition.^26^ The concentration of relaxin, produced by the fetoplacen-tal unit, increases around days 20–25 of pregnancy;^27^ from day 29 a commercially available relaxin test developed for dogs can be used for reliable pregnancy diagnosis in cats.^28^

**Figure 2:**
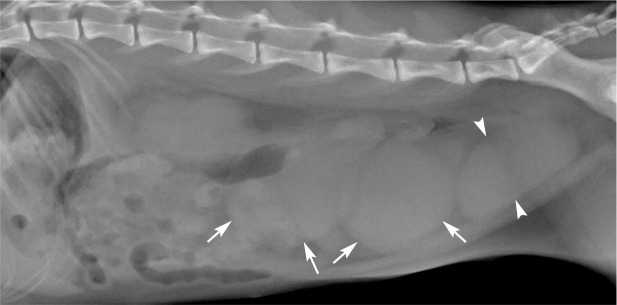
Left lateral projection of the abdomen of a 2-year-old female cat with unknown pregnancy length. Several soft tissue, opaque, lobulated structures (arrows), representing the uterus, are seen. The most caudal oval soft tissue opacity represents the urinary bladder (arrowheads). Lack of fetal mineralisation suggests <35 days’ gestation, while the lobulated shape indicates mid-pregnancy. *Courtesy of Jessica Ingman*

Pregnancy diagnosis can also be made by abdominal palpation. Though possible as early as day 15, it is easiest on days 21–25 and difficult after day 35, when the uterine swellings are confluent.^29^ Radiography can be used for pregnancy diagnosis (Figure 2), but ultrasound is more common and will also give information about the viability of the fetuses. The gestational chambers are visible from day 10, fetal heart activity from days 16–17, and an outline of the heart with chambers from day 50.^29^ Based on visible structures, the developmental stage and thus gestational age can be determined.^30^ Gestational age can also be calculated based on the diameter of the fetal abdomen or fetal stomach, or the biparietal diameter of the fetal skull.^31^ Formulas have been derived to predict parturition (days before parturition [DBP]), based on the diameter of the gestational sac (inner chorionic cavity) during the first half of pregnancy (days 19–37), and measurement of fetal biparietal diameter from day 38 (see box).^32,33^ Their accuracy is dependent on the stage of gestation, and decreases close to term.^34,35^

**Figure:**
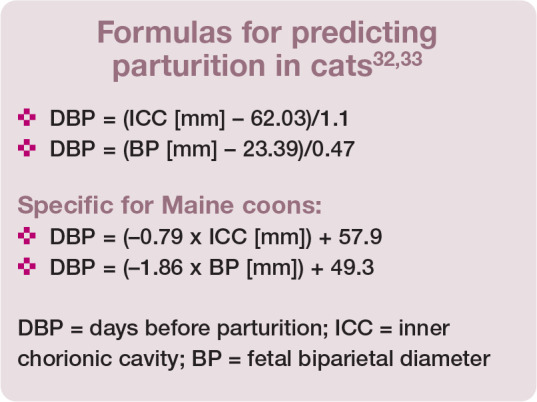


## Mammary fibroadenomatosis

Mammary fibroadenomatosis (also called fibroepithelial hyperplasia or mammary hypertrophy) is a progesterone-dependent condition characterised by proliferation of epithelium, myoepithelium and fibroblasts, and causing enlargement of one or more mammary glands.^36^ The condition may develop during pregnancy, especially in young queens,^37^ but is also seen in the non-pregnant luteal phase, including after medical treat-ments inducing ovulation, or after treatment with exogenous progestins,^38-40^ and occasionally too in male cats.^41,42^ In fibroadenomatous tissue, a strong expression of insulin-like growth factor I (IGF-I) and of receptors for progesterone and growth hormone has been described.^43^ The severity of the condition varies: sometimes milder, firm, cold swellings are detected; in other cases mastitis and abscessation may develop. Diagnosis is usually based on history and typical clinical signs. The condition often occurs only once in a queen, but it may also recur.^44^

Administration of a progesterone receptor blocker, aglepristone, is an efficient treatment for the condition, but will cause abortion in pregnant queens. Different treatment protocols have been described. Initiating treatment with 10 mg/kg SC on days 1 and 2, followed by administration once a week until remission, was effective in a study of 14 queens.^44^ A treatment period of 3-–4 weeks is often enough, but longer durations may be needed if long-acting exogenous progestins have been administered. Normal pregnancies and parturitions without relapse have been described after treatment.^44^ Ovariohysterectomy is effective if the condition is caused by endogenous progesterone production, but additional treatment with a progesterone receptor blocker may be needed in cats treated with exogenous progestins, due to persisting high progestin concentrations even after removal of the ovaries.^45^

**Figure:**
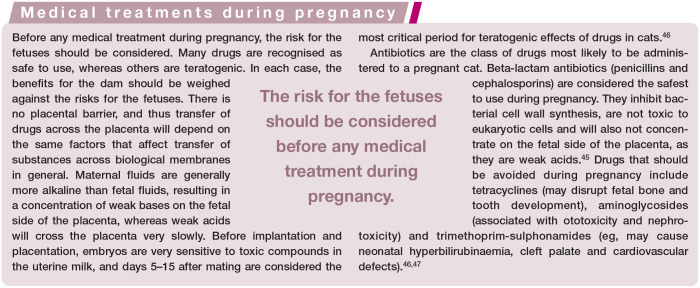


## Parturition

The mean gestation length in domestic cats is approximately 65 days (range 57–72 days), with the majority of parturitions (95-97%) occurring between 61 and 70 days.^48,49^ Parturition can be divided into three stages: the first characterised by uterine contractions and dilation of the cervix; the second by abdominal contractions accompanying productive uterine contractions and, in normal parturitions, resulting in expulsion of the fetuses; and the third involving expulsion of fetal membranes. The second and third stages often occur concurrently.

Most kittens are born in anterior position, with a smaller proportion (31% in one study^49^) born in posterior position. Stage 1 parturition usually takes less than 2 h, and stage 2 (between the birth of the first and last kitten) is usually less than 6 h, but may exceptionally be longer than 48 h.^48^ Although the time between expulsion of successive kittens may vary widely, the median time is 30 mins, and 95% of kittens are born within 100 mins of the preceding one.^49^

## Dystocia

Dystocia is a reproductive emergency that is life-threatening to both dam and kittens. The incidence of dystocia among pedigree breeding cats is typically less than 10%,^2,3,48,50^ but there is a significant variation between breeds, pointing to a genetic component.^51^ Higher incidence rates have been described in several breeds (see box below), among them the Birman;^51^ in a Finnish study, 15% of Birmans were diagnosed with dystocia.^50^ Dystocia has been associated with both small^5,48^ and large^5^ litter sizes.

Dystocia may be due to maternal and/or fetal factors. The most common cause of feline dystocia is uterine inertia, which accounts for approximately two-thirds of cases.^52^ Complete primary uterine inertia is diagnosed when there are no signs of stage 2 parturition after the due date is passed, whereas partial primary uterine inertia is diagnosed when the queen reaches stage 2 parturition but uterine contractions are weak and delivery of one or more fetuses fails. Because of the varying gestation length in cats, complete primary inertia can be difficult to diagnose. To avoid fetal mortality, caesarean section is recommended 71 days after mating if there are no signs of impending parturition.

Fetal malpresentation is considered the second most common cause of dystocia, followed by malformations, fetal death, narrow birth canal and large fetal size.^52,53^

**Figure:**
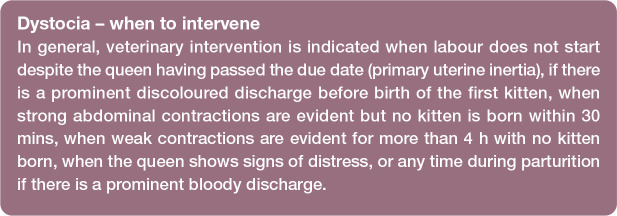


**Figure:**
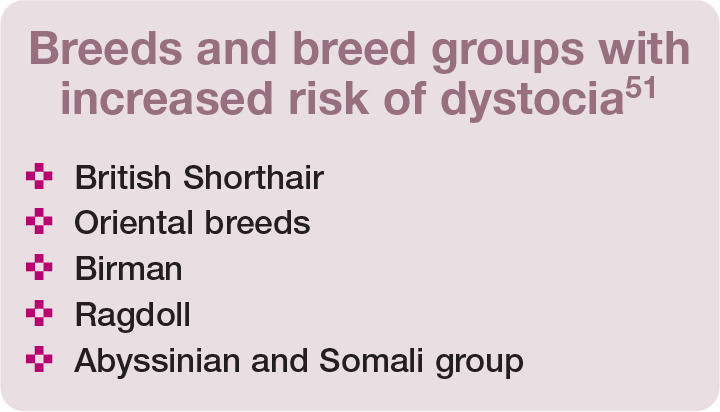


Queens with dystocia should receive a thorough assessment, comprising evaluation of general condition and a vaginal examination for the presence of fetuses. A rare cause of dystocia is uterine torsion, the twisting of a uterine horn or the uterine body about the longitudinal axis. It is an important differential in cases of dystocia, especially in queens in poor general condition. Initial supportive treatment and early surgical intervention is needed in these cases, and a definitive diagnosis can often not be made until surgery.^54^ Uterine torsion may also develop earlier during pregnancy.^55^

Radiology gives information on the number of fetuses and ultrasound allows evaluation of fetal stress and viability. A fetal heart rate >180 beats per minute (bpm) is considered normal, and below 150 bpm is an emergency. A heart rate of 150–170 bpm indicates moderate to severe fetal stress.^56^ A transient reduction in fetal heart rate may occur during exposure to a uterine contraction, and thus a fetus with a low heart rate should be monitored for a longer time (30–60 s), or the monitoring should be repeated after a few minutes, to differentiate between the effects of a uterine contraction and fetal distress.^56^

### Medical treatment

When a clinical assessment of the dam and fetuses has been performed, and dystocia has been diagnosed and characterised, treatment with ecbolic drugs may be instituted if there is no obstructive cause of the dystocia and the general condition of the dam and fetuses is good. To increase the quality and frequency of uterine contractions, oxytocin may be administered IM; initial doses of 0.1 IU/kg have been recommended, and up to 0.5-2 IU per cat.^57^ Oxytocin may also be administered IV, using the lower doses, by adding to intravenous fluids and giving slowly. Admin -istration may be repeated after 30 mins, but further administrations should be avoided due to the increased risk of uterine hyperstim-ulation and placental detachment, although this risk is reduced with lower doses.^57^

Intravenous 10% calcium gluconate may be administered slowly at 0.2 ml/kg, with cardiac monitoring, but should be avoided in compromised cardiac patients.^47^ Sub -cutaneous administration, diluted in saline, is also possible. Neither hypoglycaemia nor hypocalcaemia is common in queens with dystocia.^53^ Medical treatment of dystocia is generally considered less successful in queens than in bitches, succeeding in approximately 30% of cases.^52,53^

### Caesarean section

Although caesarean section is a common procedure in cats, and there are several reviews discussing the technique and anaesthetic con-siderations,^56-58^ there is very little scientific evidence regarding anaesthesia. When performing a caesarean section, the aim is to deliver the kittens after as short as possible exposure to the drugs. All perioperative drugs may have an effect on the kittens. Premedication with opioids is generally not necessary, and opioids may instead be given after all kittens have been delivered. if pre-medication with opioids is desired, short-acting opioids should be chosen, as these can be easily reversed in the neonate by administering naloxone on the tongue. Alfaxalone has been recommended as the agent of choice for induction,^58^ based on better neonatal viability shown in dogs.^59^ Propofol is used widely, and is an acceptable alternative.^58^ For maintenance, inhalation with isoflurane is recommended.^58^

if no more litters from the queen are desired, ovariohysterectomy may be performed following the caesarean section without negative effects on lactation. An en bloc ovariohysterecto-my, performing the ovariohysterectomy before hysterotomy and delivery of the neonates, has been described as an alternative to caesarean section that is safe for the queen.^60^ The survival rate of the kittens with this technique is comparatively low, however, and en bloc ovario -hysterectomy may best be used when the fetuses are dead, especially if there is suspicion of infectious content within the uterus.

## Uterine prolapse

**Figure 3:**
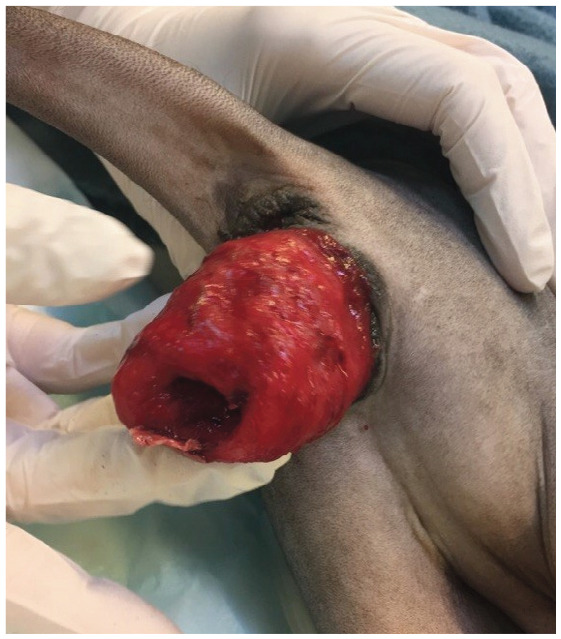
Prolapse of the uterine body with endometrial eversion in a Sphynx cat. *Courtesy of Ulrika Hermansson*

Uterine prolapses are rare reproductive emergencies in cats. They are seen at, or in the days following, parturition (both normal and associated with dystocia). The uterine body and one or both uterine horns may prolapse. Diagnosis is based on history and clinical examination. in cats with uterine prolapse, endometrial eversion is evident (Figure 3). The presence of uterine contents should be assessed by palpation or ultrasonography.

The female may be unaffected, but in some cases there is severe systemic disease, with haemorrhage and shock. if the general condition of the queen is good, treatment includes cleaning and replacement of the uterus, if it is not severely damaged. in more severe cases uterine amputation is recommended. Ovariohysterectomy is generally advised in the event of uterine prolapse, either in conjunction with prolapse replacement or at a later date. Although very rare, cases of uterine evisceration through a vaginal tear have been described, without endometrial eversion, requiring prompt surgical intervention.^62–63^

## Galactostasis and mastitis

Galactostasis and mastitis are predominantly seen during lactation, but mastitis is occasionally present during late pregnancy. Galacto-stasis may be a sequela of inadequate nursing and manifests as swollen and firm glands in a female in otherwise good health; in some cases it may progress to mastitis. Mastitis may present as a subclinical disease, with decreased weight gain in the neonates being the primary sign, but it can also be an acute and life-threatening condition with systemic signs including fever and depression. Clinically, one or more glands may be affected with typical signs of inflammation: red, swollen and warm, and with discoloured milk. In severe cases there may be abscessa-tion and development of gangrene.^64^

**Figure:**
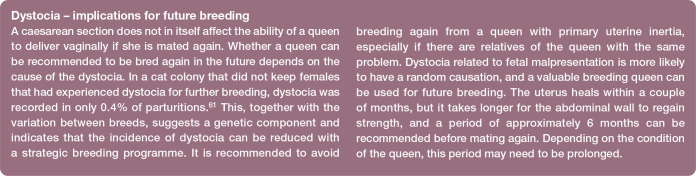


With galactostasis, massage, warm compresses and milking of affected glands may be tried. To stimulate milk release, oxytocin may be administered SC, starting at 0.5-1 IU every 30 mins. An alternative to SC administration of oxytocin is intranasal oxytocin spray, which has a short (few mins) onset of action, and may be administered into one nostril every 4–6 h.^47^ Suckling or gentle stripping is recommended to continue stimulation of milk release. Pain relief and anxiety control for the female are important.

In cases of mastitis, the choice of antimicrobial treatment will be influenced by whether there are nursing kittens, as any antimicrobial concentrating in milk will also be transferred to suckling kittens. Antibiotic selection is based on bacteriological culture and anti -microbial susceptibility testing. Because bacteria are normally present on the skin and in the teat canals, bacteriological sampling should be preceded by meticulous cleaning and disinfection of the area; the first drops should be discarded and the results interpreted with caution. The causative agent should be susceptible to the chosen antimicrobial, which should cross the blood-milk barrier and concentrate in milk without a negative influence on any suckling kittens. Anti -microbials that are weak bases will concentrate in the acidic milk.

**Figure 4:**
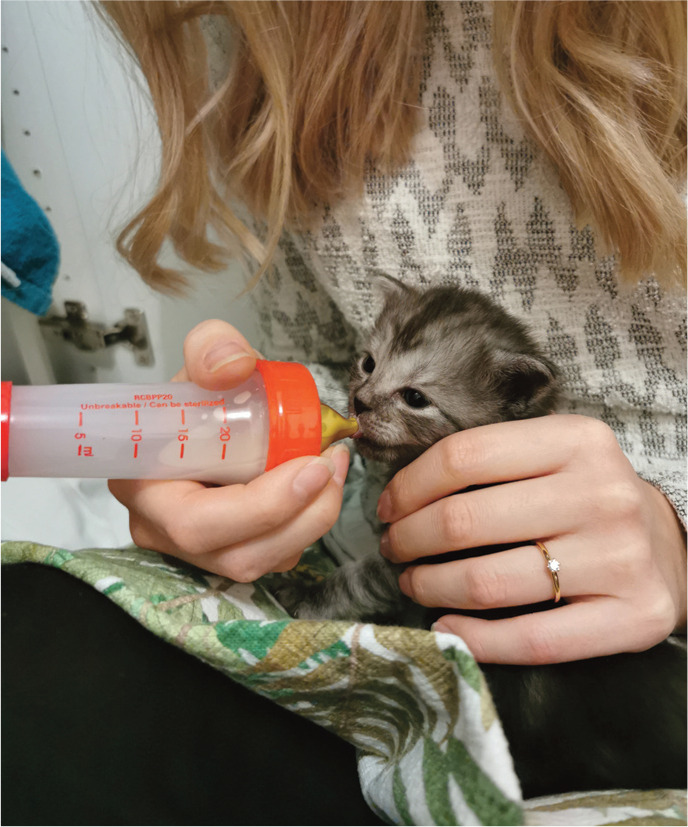
In cases of mastitis, the owner may have to remove the kittens and give milk replacement formula to avoid side effects on kittens caused by the antibiotic treatment. *Courtesy of Emma Jettel*

Empirical treatment with amoxicillin can be commenced while culture results are pending if there are nursing kittens, and with fluoro-quinolones if there are no nursing kittens and disease is severe. Note that fluoro-quinolones should only be used after susceptibility testing or in severe cases, because of the risk of bacterial multidrug-resistance developing.^65^ Extended-spectrum third and fourth generation cephalo-sporins and fluoro-quinolones are critically important drugs, and empirical use should be avoided whenever possi-ble.^65^ If fluoroquinolones, tetracyclines or chloram-phenicol are indicated, nursing kittens should be removed and given milk replacement formula (Figure 4).^47^

There is little evidence regarding how long treatment should be continued for. Depending on the severity of the condition, 7–10 days may be sufficient, but periods of 2 weeks have also been suggested.^47^ In severe cases with systemic infection, fluid therapy and intravenous antibiotic treatment is required, and abscessa-tion may be treated by surgical drainage;^37^ in such cases, nursing kittens need to be removed and cabergoline may be useful to suppress milk production.^47^

## Metritis

Metritis is a postpartum disorder. The queen is often severely depressed, with fever and a purulent vaginal discharge. Ultrasonography usually reveals a large fluid-filled uterus, and haematology shows inflammatory changes. A sample of the discharge (which likely reflects the uterine content) should be collected for bacteriological culture and susceptibility testing. *Escherichia coli* is considered the most common causal organism.

Fluoroquinolones can be recommended for critically ill queens, combined for example with ampicillin or amoxicillin, possibly potentiated with clavulanic acid, for coverage for staphylococci and streptococci. Kittens should be prevented from nursing. In less severe cases and with nursing kittens, ampicillin or amoxicillin, possibly potentiated with clavu-lanic acid, can be used until results are available from bacteriological culture.^47^

Within the first 24 h post-parturition, 0.25-1 IU IM of oxytocin will help evacuate the uterus, but after this time receptors for oxytocin are not present. Prostaglandins may be given at any time after parturition if further evacuation of contents is needed,^47^ using the low dosage regimen described below for pyometra. In severe cases, ovariohysterecto-my may be indicated.

## Pyometra

**Figure 5:**
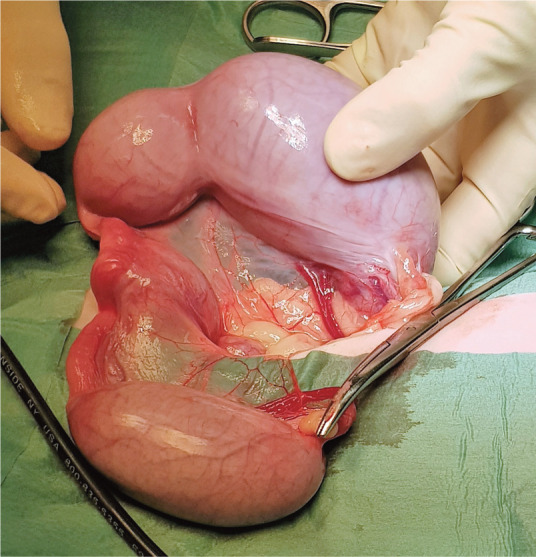
Pyometra that developed in a young Bengal cat after mating. *Courtesy of Ulrika Hermansson*

Pyometra is a life-threatening condition in cats, as in dogs. The risk increases with age and the disease is most common in middle-aged and older cats.^66^ There is a significant variation between breeds, indicating a hereditary component.^66^ Although the cat is generally considered an induced ovulator, spontaneous ovulations, followed by a luteal phase, occur regularly.^67^ Pyometra (Figure 5) is typically seen during the luteal phase,^68^ but may also be diagnosed in queens treated with progestins.^69^ Occasionally, pyometra occurs in spayed queens with ovarian remnants, usually after signs such as oestrus behaviour indicating endocrine activity.^70,71^ Rare cases may occur in conjunction with uterine neoplasia.^72^ Pyometra involves both hormonal and bacterial factors, and the most frequently isolated bacterium is *E coli.*^73^

**Figure 6:**
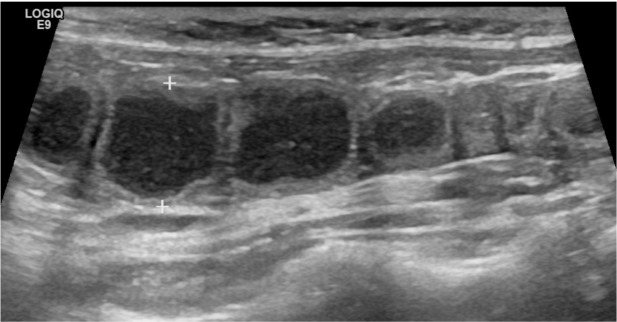
Ultrasound image obtained with an 11 MHz linear transducer in a cat with pyometra. The uterus is enlarged and tortuous, measuring approximately 1 cm between the calipers. The lumen is filled with particle-rich, hypoechoic fluid. *Courtesy of Jessica Ingman*

Common clinical signs include vaginal discharge, anorexia, lethargy, abdominal distension, pyrexia and polyuria/polydipsia.^74^ In cases of closed pyometra or in queens with meticulous cleaning habits, a vaginal discharge may not be evident. A tentative diagnosis is based on history and clinical findings, together with haematology and blood chemistry (including acute phase proteins), and ultrasonography (Figure 6) or radiology.^73^

Surgical treatment (ovariohysterectomy) is usually preferred as it is safe and effective, removing the infectious material and preventing recurrence.^73^ In breeding animals, or when anaesthesia or surgery imposes an increased risk, medical treatment is an option. Candidates for medical treatment should be selected carefully - this is not the treatment of choice for queens with serious illness. Medical treatment in breeding animals is mainly an alternative when the queen has been treated with progestins or if there are no relatives of the cat that have developed the disease, and thus the likelihood of a genetic background is low. If there is clustering of cases in related cats, further breeding should be avoided.

Because pyometra is a progesterone-dependent condition, the fundamental aim of medical treatment is to prevent the effect of progesterone. This can be achieved by administering a steroid receptor blocker, such as aglepristone.^75^ Aglepristone is considered the medical treatment of choice for pyometra and can be used also in queens with closed cervix pyometra, as the drug leads to opening of the cervix without directly causing uterine contractions; queens usually respond well to medical treatment at a dose of 10–15 mg/kg.^76,77^ A protocol involving 10 mg/kg SC aglepristone on days 1, 2 and 8, and on day 15 if needed, was successful in a study of 9/10 cats, with a follow-up period of 2 years.^77^ Queens in that study that were bred from following treatment delivered live kittens.

In countries where aglepristone is not available, the effect of progesterone can be prevented by the induction of luteolysis using prosta-glandins. Prostaglandins, natural prostaglandin F2a or synthetic cloprostenol, should only be used if the cervix is open, and with great care in animals in poor general condition due to side effects such as diarrhoea, vomiting and vocalisation, which occur 10–30 mins after treatment. Treatment with cloprostenol was effective in 5/5 queens with a follow-up period of 1 year, with two of the queens later producing litters after a subsequent mating.^78^

There is a paucity of data on the use of prostaglandins in queens. A protocol starting on the first day with a low dosage (10–15 µg/kg q6h SC) of natural prostaglandin F2α and then gradually increasing to a dosage of 50 µg/kg q8h SC by days 3–5 is now recommended to reduce side effects.^66^ Cloprostenol may also be used, gradually increasing to 1–2 µg/kg SC q12–24h. Aglepristone and prostaglandin can be used in combination. In these cases it is recommended that prostaglandin treatment starts on day 3, allowing time for aglepristone (started on day 1) to be effective in opening the cervix.^66^ Treatments with aglepristone or prosta-glandins are generally combined with trimethoprim-sulfadoxine or amoxicillin for 7 days. Fluoroquinolones penetrate uterine tissue well but, because they promote selection of multidrug-resistant bacteria, use on an empirical basis should be avoided.^65^

**Key Points**

✜ A group size of three or four cats facilitates measures against infections and decreases the risk of stress in the cats.
✜ When considering medical treatment during pregnancy, benefits for the dam should be weighed against risks for the fetuses.
✜ Culture with susceptibility testing is indicated for bacterial infections, and empirical use of fluoroquinolones reserved for severe cases.
✜ There is a breed predisposition for pyometra and certain types of dystocia, and queens that have been treated for these conditions should only be used for further breeding in cases where a genetic background is less likely.
✜ Both mammary fibroadenomatosis and pyometra are progesterone-dependent conditions that can be treated with progesterone receptor antagonists.
